# Draft Genome Sequences of *Dysgonomonas* sp. Strains BGC7 and HGC4, Isolated from the Hindgut of a Lower Termite

**DOI:** 10.1128/MRA.01427-20

**Published:** 2021-01-28

**Authors:** Charles M. Bridges, Michael C. Nelson, Joerg Graf, Daniel J. Gage

**Affiliations:** a Department of Molecular and Cell Biology, University of Connecticut, Storrs, Connecticut, USA; Indiana University, Bloomington

## Abstract

*Dysgonomonas* spp. are facultative heterotrophs which colonize diverse environments, including the hindgut of the lower termite Reticulitermes flavipes. *Dysgonomonas* genomes are enriched for genes involving oligo- and polysaccharide utilization, enabling modification of a wide array of complex glycans. Here, we report draft genome sequences for *Dysgonomonas* sp. strains BGC7 and HGC4.

## ANNOUNCEMENT

*Dysgonomonas* spp. are facultative anaerobic heterotrophs which grow on many oligo- and polysaccharides. *Dysgonomonas* spp. are particularly prevalent in lignocellulolytic termites and cockroaches (reference 1 and references therein), and there is interest in understanding their metabolic potential, particularly with regard to the biotransformation of complex lignocellulose- and host-derived glycans (2). Here, we present draft genome sequences for *Dysgonomonas* sp. strains BGC7 and HGC4, cultured from the hindgut of the lower termite Reticulitermes flavipes.

Termite hindguts were extirpated using the technique described by Matson et al. (3). Strain BGC7 was isolated on modified Eggerth-Gagnon agar (ATCC medium, 2840), and strain HGC4 was isolated on the same medium, but sterile sheep blood was replaced with a hemin-vitamin K_1_ solution (25 mg and 5 mg ml^−1^, respectively). Cultures were maintained on rich peptone-hemin-glucose (rPHG) agar (1), and plates were grown anaerobically under an atmosphere of 89% N_2_, 5.5% CO_2_, and 5.5% H_2_ at 22°C. 16S rRNA gene sequences from isolates BGC7 and HGC4 (1) (available under GenBank accession numbers MT340878 and MT340881, respectively) placed both isolates within the genus *Dysgonomonas*. Genomic DNA was prepared using a Promega Wizard genomic DNA purification kit. Concentration and quality were determined by using gel electrophoresis and a Qubit fluorometer. DNA was mechanically sheared and Illumina TruSeq PCR-free libraries were prepared using DNA size selected for 550 bp. Libraries were sequenced on an Illumina MiSeq instrument using a 2 × 250-bp v2 kit. BBTools v36.38 (https://sourceforge.net/projects/bbmap/) was used to remove adapter sequences and quality trim and filter raw reads (threshold of Q15). Processed reads were assessed with FastQC v0.11.5 (https://www.bioinformatics.babraham.ac.uk/projects/fastqc/), and draft assemblies were created using A5-miseq v20160825 (4) and SPAdes v3.12.0 (5) using default settings on the KBase platform (6). Assemblies were merged using MAC (19 July 2020) (7) with default settings. Processed reads were aligned to MAC-merged assemblies using Bowtie 2 v2.3.2 (8), and SAMtools v1.10 (9) was used for file conversion. Pilon v1.23 (10) was used to polish merged assemblies iteratively three times. Polished assemblies were manually curated and annotated by submission to the NCBI Prokaryotic Genome Annotation Pipeline (PGAP) v4.13 (11). Genomes were checked for completeness and contamination using CheckM v1.0.18 (12) on KBase. Genomic relatedness to type and non-type strain *Dysgonomonas* genomes (available from RefSeq as of 15 October 2020) was determined by digital DNA-DNA hybridization (dDDH) performed using the Type Strain Genome Server (13). Average amino acid identities (AAI) between orthologous genes were computed with CompareM v0.1.1 (https://github.com/dparks1134/CompareM). Carbohydrate active enzyme (CAZy) domains were identified using the dbCAN2 server (14), and polysaccharide utilization loci (PULs) were identified using PULpy (15), with both using default settings.

Genome accession details, assembly metrics, phylogenetic relatedness, and CAZy/PUL content can be found in Table 1. The genomes of *Dysgonomonas* sp. strains BGC7 and HGC4 were enriched for CAZy domain-containing proteins and PULs involved in degradation of plant polysaccharides, particularly xylans. The availability of these sequences will contribute to understanding the metabolic potential and diversity of lignocellulose-degrading organisms, particularly within the genus *Dysgonomonas*.

**TABLE 1:** Genome details of *Dysgonomonas* sp. strains

Parameter	Data for strain:	
	BGC7	HGC4
BioProject accession no.	PRJNA656570	PRJNA656570
BioSample accession no.	SAMN15793200	SAMN15793201
SRA accession no.	SRR12430954	SRR12430953
WGS accession no.	JACMIC000000000.1	JACMIB000000000.1
RefSeq accession	GCF_014840225.1	GCF_014840235.1
Taxonomy ID^*a*^	1658008	1658009
No. of raw sequencing reads	4,689,088	4,276,966
No. of filtered reads	4,265,276	3,081,766
Mean scaffold coverage^*b*^ (×)	223	154
No. of scaffolds >100 bp	25	18
No. of contigs >100 bp	32	22
Largest scaffold (bp)	1,432,443	856,340
Genome size (bp)	4,336,043	4,502,296
*N*_50_ (bp)	874,238	769,148
*L*_50_ (bp)	2	3
G+C content (%)	37.13	35.92
No. of coding sequences	3,507	3,644
No. of protein-coding sequences	3,451	3,601
No. of tRNAs	42	42
Completeness (%)	100	100
Contamination (%)	0.55	0
Closest neighbor, 16S rRNA gene (% identity)	Dysgonomonas gadei ATCC BAA-286^*c*^ (96.42)	*Dysgonomonas* sp. HDW5A^*d*^ (98.42)
Closest neighbor, whole genome (% dDDH^*e*^)	Dysgonomonas capnocytophagoides DSM 22835^*f*^ (26.5)	Dysgonomonas alginatilytica DSM 100214^*g*^ (34.1)
Closest neighbor, protein-coding sequences	*D. gadei* ATCC BAA-286^*c*^	*D. alginatilytica* DSM 100214^*g*^
Orthologous fraction of genes^*h*^ (% of total)	65.43	78.09
AAI between orthologs^*i*^ (%)	75.35	90.44
Total no. of CAZy domains^*j*^	209	282
No. of glycosyl hydrolase domains	149	199
No. of carbohydrate esterase domains	10	11
No. of polysaccharide lyase domains	14	9
No. of carbohydrate binding module domains	3	11
No. of glycosyl transferase domains	33	52
No. of protein-coding sequences with ≥1 CAZy domain (% of total)	207 (6.0)	267 (7.4)
Total no. of PULs	45	41
No. of CAZy-associated PULs (% of total)	29 (64)	32 (78)

a ID, identifier.

b Determined using unpaired filtered reads mapped to Pilon-polished assembly using Bowtie 2.

c RefSeq assembly accession number GCF_000213555.1.

d RefSeq assembly accession number GCF_011299555.1.

e Digital DNA-DNA hybridization values calculated using formula d_4_ (sum of all identities found in high-scoring pairs [HSPs] divided by overall HSP length).

f RefSeq assembly accession number GCF_000426485.1.

g RefSeq assembly accession number GCF_003201355.1.

h The number of orthologs divided by the minimum number of protein-coding genes from either genome.

i Average amino acid identity between orthologous fraction of genes.

j Domains identified by HMMER and at least one other tool (HotPep or DIAMOND).

### Data availability.

Genome assemblies for isolates BGC7 and HGC4 were submitted to NCBI under BioProject accession number PRJNA656570 and whole-genome sequence (WGS) accession numbers JACMIC000000000.1 and JACMIB000000000.1, respectively. Detailed information can be found in Table 1.
